# Response inhibition deficits are positively associated with trait rumination, but attentional inhibition deficits are not: aggressive behaviors and interpersonal stressors as mediators

**DOI:** 10.1007/s00426-021-01537-y

**Published:** 2021-06-06

**Authors:** Akira Hasegawa, Noboru Matsumoto, Yuko Yamashita, Keisuke Tanaka, Jun Kawaguchi, Tetsuya Yamamoto

**Affiliations:** 1 Faculty of Human Relations, Tokai Gakuin University, 5-68 Naka-kirino, Kakamigahara City, Gifu 504-8511 Japan; 2 Division of Psychology, Faculty of Arts, Shinshu University, Asahi 3-1-1, Matsumoto, Nagano 390-8621 Japan; 3 Mutsumi Hospital, 3-11-23, Minamiyaso-cho, Tokushima, 770-0005 Japan; 4 Graduate School of Education, Joetsu University of Education, 1-Yamayashiki-machi, Joetsu-shi, Niigata, 943-8512 Japan; 5 Department of Psychology, Otemon Gakuin University, 2-1-15, Nishiai, Ibaraki City, Osaka 567-8502 Japan; 6 Graduate School of Informatics, Nagoya University, Furo-cho, Chikusa-ku, Nagoya, Aichi 464-8601 Japan; 7 Graduate School of Technology, Industrial and Social Sciences, Tokushima University, 1-1, Minamijosanjima-cho, Tokushima, 770-8502 Japan

## Abstract

Previous findings on relationships between inhibition that is a core executive function, and trait rumination have been inconsistent. This inconsistency could be overcome by investigating the association between rumination and the two subcomponents of inhibition: response inhibition and attentional inhibition. This study examined whether and how response inhibition and attentional inhibition were related to rumination as well as worry. University students in Japan (*N* = 213) conducted the Go/No-Go Task and the Modified Stroop Task. They also completed self-report measures of depression, trait rumination, trait worry, stressors, and aggressive behaviors. Results indicated that response inhibition deficits were positively associated with trait rumination, and this association was mediated by increases in aggressive behaviors and interpersonal stressors. The associations between these variables remained significant even after controlling for depression level. There were no significant direct or indirect associations between attentional inhibition deficits and rumination. These results suggest that response inhibition deficits, among the subcomponents of inhibition, have an indirect positive association with rumination through interpersonal processes. Results also showed nonsignificant differences between rumination and worry in the magnitude of correlation coefficients with the two subcomponents of inhibition. Therefore, it remains unclear whether the positive association with response inhibition is unique to rumination.

## Introduction

Rumination is repetitive and passive thinking about one’s depressive symptoms and possible causes and consequences of these symptoms (Nolen-Hoeksema, 1991; Watkins & Roberts, 2020). Previous studies suggested that rumination is a core vulnerability to depression. For example, dysphoric and depressed individuals who are induced to ruminate exacerbate their depressive mood (Donaldson & Lam, 2004; Nolen-Hoeksema & Morrow, 1993). In addition, trait rumination, which is assessed using the total score on the Ruminative Responses Scale (RRS; Nolen-Hoeksema & Morrow, 1991), predicts more severe depression (Butler & Nolen-Hoeksema, 1994; Nolan et al., 1998; Nolen-Hoeksema & Morrow, 1991), and the onset of major depression (Nolen-Hoeksema, 2000; Nolen-Hoeksema et al., 2007; Spasojević & Alloy, 2001). Previous studies have also indicated that rumination leads to deleterious consequences such as increases in associated negative thinking, ineffective social problem solving, interference with active instrumental behaviors, and reduced sensitivity to changing contingencies and contexts (for reviews, Watkins & Roberts, 2020).

During the past 2 decades, researchers have examined the possible role of executive function deficits in increasing rumination. Executive function is a family of top–down control processes that are needed when going automatic, or relying on instinct or intuition would be ill-advised, insufficient, or impossible (Diamond, 2013). Previous meta-analyses have suggested that deficits in executive functions were positively associated with trait rumination (Yang et al., 2017; Zetsche et al., 2018). However, no definite conclusions have been drawn to date on the subcomponents of executive function that could be related to increased rumination. Notably, the performance in tasks assessing inhibition has shown inconsistent associations with trait rumination, as described below.

Executive function tasks used in psychopathology research have involved emotional and non-emotional stimuli. The current study focused on executive function tasks using non-emotional stimuli because performance in emotional tasks might reflect not only impairments in executive function but also differences in emotional processing (Snyder et al., 2015).

Inhibition is the ability to deliberately inhibit dominant, automatic, or prepotent responses when necessary (Miyake et al., 2000). Several studies have examined the association between trait rumination and performance in emotionally neutral inhibition tasks. A previous study by Hasegawa et al. (2021) assessed the inhibition of prepotent behaviors and reported a significant positive association between trait rumination and inhibition deficits. The authors constructed a latent variable from the performance in the Go/No-Go Task (GNG), the Stop Signal Task (SST), and the Conners’ Continuous Performance Test 3rd Edition (CCPT) and examined the association between this latent variable and trait rumination in university students. Their results indicated that the latent variable reflecting deficits in inhibition of prepotent behaviors had a positive indirect association with rumination via increased stressors. These findings suggest that the impaired ability to inhibit prepotent behaviors could lead to problematic behaviors, and resulting stressors might, in turn, increase rumination.

In contrast, a contradictory finding was obtained in an initial study that examined associations between trait rumination and performance in the Modified Stroop Task (MST), which is a popular measure of inhibition. Altamirano et al. (2010) indicated that ruminative undergraduate students perform *better* in the MST, as indicated by fewer errors in incongruent trials, and this association was significant even after controlling for the participants’ depression level. They interpreted this result as reflecting higher active maintenance of a single task goal by trait ruminators in the face of distractions. The findings by Altamirano et al. (2010) contradict the assumption that high ruminators have inhibition impairments, although caution is needed because a subsequent study found no significant correlation between trait rumination and MST performance (Nishimura et al., 2020).

These inconsistent results could be explained by a recent, empirically supported model of inhibition. Tiego et al. (2018) proposed that inhibition could be divided into two subcomponents: response inhibition representing the ability to suppress a prepotent motor response, and attentional inhibition representing the ability to resist interference from distracting stimuli. They conducted a latent variable analysis using three tasks that were assumed to assess response inhibition and three tasks assessing attentional inhibition and reported data supporting their model, in which each subcomponent of inhibition is distinguished from the other. According to this classification, the GNG, the SST, and the CCPT were clarified as tasks assessing response inhibition, and the MST was clarified as a task assessing attentional inhibition. The inconsistent association between performance in these tasks and trait rumination in previous studies suggest that these two subcomponents of inhibition have different effects on trait rumination.

However, previous studies' findings are limited because they did not compare the relationships of response inhibition and attentional inhibition with trait rumination in the same study and possibly reporting contradictory effects of response inhibition and attentional inhibition on rumination due to differences in sample characteristics. In addition, previous studies did not adequately consider the pathways from each subcomponent of inhibition to rumination. For example, although Hasegawa et al. (2021) showed that a latent variable that reflected response inhibition deficits was indirectly associated with increased trait rumination via increased stressors, they did not examine the behaviors that mediated the association between response inhibition and stressors. Moreover, previous studies have not examined the possible mediators in the association between attentional inhibition and rumination (Altamirano et al., 2010; Nishimura et al., 2020).

The present study’s primary purposes were to compare the concurrent association of response inhibition and attentional inhibition with trait rumination and to examine possible pathways from these subcomponents of inhibition to rumination in university students. Following Hasegawa et al. (2021), we assumed that response inhibition deficits were positively associated with rumination via increased stressors. We also examined whether aggressive behaviors mediated the association between response inhibition and interpersonal stressors because previous studies have shown that aggressive behaviors were one behavioral correlate of impaired response inhibition (Qiao et al., 2016; Raaijmakers et al., 2008). On the other hand, following Altamirano et al. (2010), we assumed that attentional inhibition deficits were negatively associated with rumination. We used the GNG as an index of response inhibition and the MST as an index of attentional inhibition because previous studies have demonstrated that performance in these tasks is significantly related to trait rumination (Altamirano et al., 2010; Hasegawa et al., 2021).

Previous studies have examined whether depression confounded the relationship between rumination and other factors related to rumination, such as executive functions (see Zetsche et al., 2018). Therefore, we also investigated whether the relationships among each inhibition subcomponent, aggression, stressors, and rumination remained significant, even after controlling for the influence of depressive symptoms.

We also examined models that replace rumination assessed with the total RRS scores with brooding and reflection subcomponents of rumination. Brooding is “a passive comparison of one’s current situation with some unachieved standard,” and reflection is “a purposeful turning inward to engage in cognitive problem solving to alleviate one’s depressive symptoms” (Treynor et al., 2003, p. 256). Previous studies have suggested that brooding and reflection were distinct subcomponents because brooding was more strongly associated with concurrent and future depression levels than reflection (Pearson et al., 2010; Schoofs et al., 2010; Treynor et al., 2003). Hasegawa et al. (2021) demonstrated that the relationship between brooding and stressors was stronger than that between reflection and stressors. Therefore, it is plausible that the indirect effects of response inhibition on brooding via negative achievement and interpersonal events would be stronger than on reflection.

Finally, this study examined the relationships between trait worry and the two subcomponents of inhibition. Worry is defined as “a chain of thoughts and images, negatively affect-laden and relatively uncontrollable. The worry process represents an attempt to engage in mental problem solving on an issue whose outcome is uncertain but contains the possibility of one or more negative outcomes” (Borkovec et al., 1983, p. 10). Worry is a core feature of Generalized Anxiety Disorder (American Psychiatric Association, 2013). Rumination and worry share many characteristics, such as negative content and a perseverative style. However, rumination and worry are different because the former concerns the past, whereas the latter concerns the future (Watkins et al., 2005). Although previous studies have compared the associations of rumination and worry with executive functions, further investigations are necessary because subcomponents of executive functions that are specifically related to rumination or worry have not been identified (Zetsche et al., 2018 for meta-analysis). Therefore, the present study compared the associations of rumination and worry with response inhibition and attentional inhibition.

## Methods

### Participants

Undergraduate and graduate students aged 18–30 years were recruited from the Joetsu University of Education, Nagoya University, Tokai Gakuin University, and Tokushima University in Japan. Since prior studies have not examined the association of response inhibition and attentional inhibition with aggression, negative events, and rumination after controlling for other subcomponents of inhibition, we could not decide the sample size or power for this study based on prior studies. As a result, we attempted to collect as much data as possible within 1 year using available resources. Two hundred and thirteen students (81 men and 132 women; mean age = 19.76, SD = 1.44; age ranged from 18 to 26 years) participated in this study. We recruited students in this age range to avoid any age effects. All participants were Japanese, with the exception of one participant who was Vietnamese.

### Self-report measures

*Beck Depression Inventory-Second Edition* (BDI-II; Beck et al., 1996): The BDI-II is a well-validated questionnaire assessing the severity of depressive symptoms experienced in the past 2 weeks. Participants respond to 21-item using a 0–3 scale, with higher scores indicating more severe depression. The Japanese translation of the BDI-II by Kojima and Furukawa (2003) was used in this study. The BDI-II showed excellent internal consistency (*α* = 0.90) in our sample.

*Ruminative Responses Scale* (RRS; Nolen-Hoeksema & Morrow, 1991): The RRS is a measure of rumination and its subcomponents. We used the Japanese translation of the RRS by Hasegawa (2013). This scale is composed of 22 items, each of which is rated on a 4-point rating scale anchored between 1 (*almost never*) and 4 (*almost always*). The RRS is composed of 5 items assessing brooding, 5 items assessing reflection, and 12 depression-related items. Confirmatory factor analysis of the data indicated that the two-factor model comprising of brooding and reflection had an acceptable fit indices range (*χ*^2^ (34) = 87.579, *p* < 0.001; CFI = 0.909; RMSEA = 0.086, 90% CI: [0.064, 0.108]). The fit indices of the two-factor model were insufficient (Hu & Bentler, 1998). Nevertheless, there was a moderate correlation between brooding and reflection (*r* = 0.477; see Table 2), indicating that these two subscales assessed distinct components of rumination. These two RRS subscales have been used in many previous studies (Watkins & Roberts, 2020). Therefore, we calculated and analyzed the brooding and reflection subscale scores and the total RRS score.

Adequate psychometric properties of the RRS, including good internal consistency and construct validity, as well as moderate test–retest reliability of the total and subscale scores, have been reported (Hasegawa, 2013; Schoofs et al., 2010; Treynor et al., 2003). Good internal consistencies for overall RRS (*α* = 0.92), brooding (*α* = 0.82), and reflection (*α* = 0.71) subscales were obtained in our sample.

*Penn State Worry Questionnaire* (PSWQ; Meyer et al., 1990): The PSWQ is a scale assessing the frequency and intensity of worry. This scale includes 16 items, each rated on a five-point rating scale anchored between 1 (*not at all typical of me*) and 5 (*very typical of me*). Meyer et al. (1990) demonstrated that the PSWQ has high internal consistency, high test–retest reliability, and good convergent and discriminant validity. The Japanese translation of the PSWQ by Sugiura and Tanno (2000) was used in this study. The internal consistency of the PSWQ was excellent in the present study (*α* = 0.93).

*Scale of Life Events in Interpersonal and Achievement Domains* (Takahira, 1998): This scale assesses positive and negative events in interpersonal and achievement domains that students might experience in their daily lives. The subscales assessing negative interpersonal events and negative achievement events, each consisting of 15 items, were used in this study. The negative interpersonal events subscale includes items such as “I quarreled with a family member, friend, or romantic partner” and “I was criticized or teased by friends or associates.” The negative achievement events subscale includes items such as “I got bad grades in my exams or reports” and “there were many tasks such as reports that should come to grips with.” Participants were asked to indicate how often they encountered each event during the last three months on a 4-point scale anchored between 1 (*not at all*) and 4 (*often*). We calculated scores on the negative interpersonal events subscale and negative achievement events subscale. We obtained adequate internal consistencies for negative interpersonal events (*α* = 0.82) and negative achievement events (*α* = 0.79) subscales in our sample.

*Aggression Scale* (Isobe & Hishinuma, 2007): This scale is a measure of aggression that is composed of 23 items, including four filer items. Each item was rated on a five-point rating scale using the anchors 1 (*not at all true of me*) and 5 (*extremely true of me*). This study used the overt aggression subscale score as an index of aggressive behaviors. This subscale is composed of 12 items assessing physical and verbal aggressive behaviors, including, “sometimes I was violent unintentionally,” and “sometimes I was sarcastic and said bad things to others’ faces.” Overt aggression subscale demonstrated good internal consistency (*α* = 0.84).

### Behavioral measures

*Go/No-Go Task* (GNG): We used the GNG task, which was based on the procedure adopted by Gutiérrez-Cobo et al. (2017), and Hasegawa et al. (2019). This task is composed of go trials in which a white circle with a diameter of approximately 5 cm appears in the center of a black screen and no-go trials in which a red circle of the same size appears. The participants were instructed to press the “b” key as quickly as possible in the go trials and not press any key in the no-go trials. The GNG began with the presentation of a fixation cross for 1000 ms, followed by a blank screen for 2000 ms. Then, the go and the no-go trials were presented in random order.

Participants completed a practice phase composed of 10 trials with an equal number of go and no-go trials before the test trial phase. During the practice trials, the stimulus appeared for 500 ms, with a 1000 ms inter-stimulus interval (ISIs), during which time the participants could respond. Then, participants completed the test trial phase that was composed of 4 blocks of 120 trials. The stimulus appeared for 250 ms during each trial in all the blocks. However, the ISIs varied across blocks such that the ISI in a given block was 400, 600, 800, or 1000 ms. The order of the blocks was randomized among participants. Go-trials were 70%, and no-go trials were 30% in each block. The erroneous response rate of each participant in no-go trials (commission error rate) was calculated for each block.

To measure response inhibition, we used the GNG composed of four blocks in which the ISIs differed from block to block and assessed the appropriate ISI of the GNG (this examination was unrelated to this report, and is reported in Hasegawa et al., 2020). Commission errors in blocks with 400 and 600 ms ISIs had a relatively normal distribution (Skewness ≤ 1.43, Kurtosis ≤ 2.14) compared to the skewed distributions of commission errors in blocks with 800 and 1000 ms ISIs (Skewness ≥ 2.09, Kurtosis ≥ 6.52). In addition, commission errors in the block with 600 m ISI showed a stronger positive correlation with commission errors in the Conners Continuous Performance Test 3rd Edition (CCPT; Conners, 2014) than that of 400 ms ISI. Therefore, the 600 ms ISI was the optimal ISI for assessing individual differences in response inhibition among the four ISIs used in this study (Hasegawa et al., 2020). Consequently, we used the commission error rate with a 600 ms ISI as the index of response inhibition deficits.^1^ The commission error rate in this block had acceptable split-half reliability (*r* = 0.696, *p* < 0.001, 95% CI: [0.626, 0.765]).

*Modified Stroop Task* (MST): The MST based on the procedure adopted by Altamirano et al. (2010) was used. Three, 70-point Kanji (pictographic Japanese writing) characters, written in blue, yellow, and red, were used. Each character was consecutively displayed in blue, yellow, or red on each trial at the center of a black screen. Participants were instructed to press the “1” key as quickly as possible if the character’s color on the screen was blue, and “2” when the color was yellow, and “3” when the color was red, regardless of the meaning of the displayed character. The character on the screen disappeared when participants responded, and the next character appeared after an ISIs of 500 ms. This task comprised 75% congruent trials in which the color and the meaning of characters on the screen matched, and 25% incongruent trials in which they did not. The task began with the presentation of a fixation cross for 1000 ms, followed by a blank screen that was presented for 2000 ms. Next, the congruent and incongruent trials were presented in random order. The participants completed four blocks of 48 trials after the practice phase that was composed of 24 trials. The error response rate was calculated for incongruent trials in the test phase. The incongruent trials’ error rate was used for assessing attention inhibition deficits. The error rates in the incongruent trials demonstrated acceptable split-half reliability (*r* = 0.651, *p* < 0.001, 95% CI: [0.573, 0.729]).

### Procedure

Students interested in this study were individually invited to the authors’ laboratory. First, we obtained their informed consent for participating in the study. Then, participants completed the GNG, the MST, and the CCPT.^2^ All the tasks were administered using a computer screen with 1366 × 768 pixels. The GNG and the MST were administered via Inquisit 5 (Millisecond Software, LLC.). Participants could take a short break between tasks and task blocks. The participants responded to the self-report measures described above and the Japanese version of the Buss-Perry Aggression Questionnaire (BAQ; Ando et al., 1999) after completing all the tasks.^3^ They were debriefed after completing the study and were given a gift certificate worth 1500 yen (approximately 14 US dollars). The study took approximately 1 h for a participant to complete. The Ethics Committee of Tokai Gakuin University approved this study.

### Statistical analysis

Analyses were conducted on raw data and allowed for missing data. Descriptive statistics were identified and the missing completely at random (MCAR) test was conducted using SPSS ver. 23 (IBM Corporation), and the *Z*-test was conducted using R. Other analyses were conducted using Mplus 8.3 (Muthén & Muthén, 1998–2017). Zero-order Pearson’s correlations were computed between each measure, and the comparison of two dependent correlations based on dependent groups was conducted using the procedure described by Hittner et al. (2003). We conducted path analyses to examine the assumed model. The maximum number of iterations and convergence criterion in Mplus was used as defaults (i.e., 1000 iterations and 0.00005, respectively) for conducting the correlation and path analyses. In addition to the *χ*^2^, we used the comparative fit index (CFI) ≥ 0.95 and root-mean-square error of approximation (RMSEA) ≤ 0.06 as indicators of good fit (Hu & Bentler, 1998).

Of all the data, the MST data of four participants were excluded because three of these participants responded with the characters’ content instead of their colors, and one participant fell asleep during the task.^4^ Missing data patterns were examined using all the variables that were analyzed in this report that are listed in Table 1. Little’s (1988) MCAR test yielded a significant chi-square value (*χ*^2^ (52) = 77.22, *p* = 0.013), indicating that missing values were not random. However, it is unlikely that the Full Information Maximum Likelihood (FIML) method’s estimates could be highly biased because there were very few missing data points (see Table 1). Therefore, we handled missing data points in the correlation and path analyses using the FIML method. The distribution of specific variables was slightly skewed (see Table 1). Therefore, the bootstrapped standard errors were computed using 10,000 bootstrap re-samples to determine the significance of each standardized partial coefficients and indirect effects in the path analysis.

**Table 1:** Descriptive statistics of study measures

	*n*	*M*	SD	Range	Skewness	Kurtosis
Depression	212	12.45	9.34	0.00–51.00	1.15	1.45
Brooding	211	11.47	4.12	5.00–20.00	0.24	− 0.86
Reflection	212	9.39	3.40	5.00–20.00	0.73	0.01
Rumination total	210	45.39	13.53	23.00–85.00	0.38	− 0.47
Worry	213	53.42	13.73	21.00–80.00	− 0.36	− 0.76
Negative interpersonal events	213	25.64	7.12	15.00–50.00	1.01	0.78
Negative achievement events	210	27.54	6.93	15.00–53.00	0.90	1.26
Aggressive behaviors	212	23.44	7.83	12.00–56.00	1.28	2.04
Response inhibition deficits	213	13.34	12.22	0.00–63.89	1.43	2.14
Attentional inhibition deficits	209	11.07	9.33	0.00–52.08	1.49	2.91

Multivariate outliers were checked using Cook’s *D* values of each correlation among all study variables. One participant showed five correlations, two participants showed four correlations, and two participants showed one correlation indicated by Cook’s *D* values greater than one. The correlation coefficients did not differ largely when these observations were removed, except for the significant correlation between depression and attentional inhibition deficits, and the correlation between total RRS score and aggressive behaviors, which disappeared (the correlation coefficients changed from 0.174 to 0.083 in the former and from 0.165 to 0.114 in the latter). In addition, the results of path analyses conducted in this study were approximately similar to those described below, even when the data of the five participants showing Cook’s *D*s greater than one in either correlation were excluded. Therefore, we analyzed the data of all the participants.

As described above, we assumed that response inhibition was indirectly associated with rumination via aggressive behaviors and interpersonal events. We also examined the direct effects of response inhibition on both types of events because response inhibition might be indirectly related to negative interpersonal and achievement events via behaviors other than aggressive behaviors. Moreover, we examined the direct effect of attentional inhibition on rumination following the suggestion by Altamirano et al. (2010). The current study focused on comparing direct and indirect effects of response inhibition and attentional inhibition on rumination. Therefore, we examined the direct effects of attentional inhibition on aggressive behaviors and two kinds of negative events, as well as the effects of response inhibition on rumination. A correlation between two kinds of inhibition functions and that between two kinds of stressors were allowed. We also conducted path analysis with depression as an exogenous variable influencing aggressive behaviors, stressors, and rumination because depression might be a confounding factor in the relationship between rumination and other factors including executive functions (see Zetsche et al., 2018).

Specific variables of the models might be unsuitable for constructing a latent variable in these analyses. Snyder et al. (2019) indicated that negative events scales assess the frequency of different events rather than different indicators of a particular construct. Therefore, it would not be appropriate to model a latent variable of negative events by assuming that a latent construct causes frequency of each events. Furthermore, it would be easy to compare the present findings and previous findings using manifest variables because previous studies on rumination have generally used manifest variables as indicators of each construct when a single scale was used to assess each construct (Hamilton et al., 2017; Sanchez-Lopez et al., 2019; Stroud et al., 2018). Therefore, we only used manifest variables for path analyses and other analyses.

## Results

Descriptive statistics of each measure are shown in Table 1, and correlations between each measure are displayed in Table 2. Response inhibition deficits showed significant positive correlations with all study variables except worry, whereas attentional inhibition deficits had significant positive correlations with depression and response inhibition deficits. Aggressive behaviors showed significant positive correlations with brooding, total RRS scores, and negative interpersonal events.^5^ The *Z* test for comparing two dependent correlations showed that response inhibition was more strongly correlated with rumination than attentional inhibition (*z* = 2.475, *q* = 0.208, *p* = 0.014). Similarly, *Z* tests compared correlations of rumination and worry with the two inhibition measures. Results indicated that neither the correlation with response inhibition deficits nor the correlation with attentional inhibition deficits differed significantly between rumination and worry (*z* = 1.740, *q* = 0.116, *p* = 0.082; *z* = 0.635, *q* = 0.042, *p* = 0.526).

**Table 2:** Correlations between variables

	1	2	3	4	5	6	7	8	9
1. Depression	−								
2. Brooding	0.439^***^	−							
	[0.330, 0.547]								
3. Reflection	0.221^**^	0.477^***^	−						
	[0.093, 0.349]	[0.373, 0.581]							
4. Rumination total	0.526^***^	0.874^***^	0.726^***^	−					
	[0.429, 0.624]	[0.842, 0.906]	[0.663, 0.790]						
5. Worry	0.459^***^	0.523^***^	0.288^***^	0.540^***^	−				
	[0.352, 0.565]	[0.425, 0.622]	[0.164, 0.411]	[0.443, 0.636]					
6. Negative interpersonal events	0.441^***^	0.387^***^	0.198^**^	0.374^***^	0.271^***^	−			
	[0.333, 0.550]	[0.272, 0.502]	[0.069, 0.328]	[0.257, 0.490]	[0.147, 0.395]				
7. Negative achievement events	0.492^***^	0.274^***^	0.144^*^	0.290^***^	0.281^***^	0.564^***^	−		
	[0.390, 0.595]	[0.148, 0.400]	[0.012, 0.276]	[0.166, 0.414]	[0.157, 0.406]	[0.471, 0.656]			
8. Aggressive behaviors	0.083	0.159^*^	0.130	0.165^*^	− 0.077	0.211^**^	0.041	−	
	[− 0.052, 0.218]	[0.027, 0.291]	[− 0.002, 0.262]	[0.034, 0.297]	[− 0.211, 0.056]	[0.081, 0.341]	[− 0.098, 0.179]		
9. Response inhibition deficits	0.220^**^	0.178^**^	0.145^*^	0.225^**^	0.112	0.204^**^	0.195^**^	0.185^**^	−
	[0.092, 0.348]	[0.048, 0.308]	[0.013, 0.276]	[0.097, 0.352]	[− 0.021, 0.245]	[0.076, 0.333]	[0.066, 0.325]	[0.054, 0.316]	
10. Attentional inhibition deficits	0.174^**^	− 0.004	− 0.020	0.021	0.063	0.028	0.098	− 0.006	0.266^***^
	[0.043, 0.305]	[− 0.141, 0.132]	[− 0.159, 0.118]	[− 0.118, 0.160]	[− 0.071, 0.198]	[− 0.108, 0.164]	[− 0.038, 0.234]	[− 0.154, 0.143]	[0.138, 0.393]

Numbers in parentheses indicate 95% confidence intervals. * *p* < 0.05, ** *p* < 0.01, *** *p* < 0.001

**Fig. 1:**
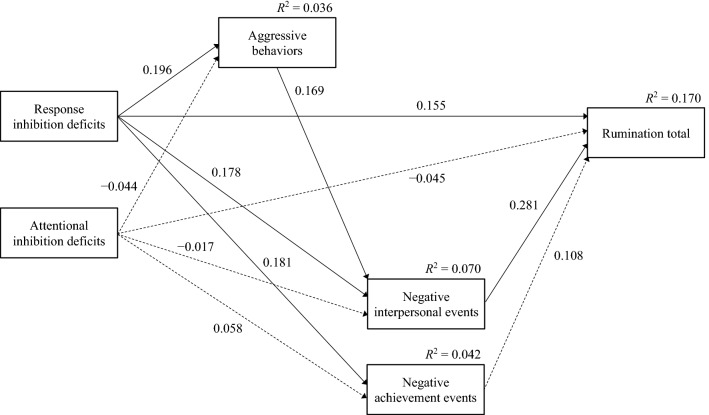
Path model examining the assumed model (*N* = 213). Significant paths are shown as solid lines (*p* < 0.05), and nonsignificant paths are shown as dashed lines. All values except *R*^2^ are standardized regression coefficients. Error variables and covariances are omitted

We conducted path analysis with FIML to examine whether and how response inhibition and attentional inhibition deficits were related to rumination assessed with the total RRS scores. The model provided an excellent fit to the data (*χ*^2^ (2) = 1.530, *p* = 0.466; CFI = 1.000; RMSEA = 0.000, 90% CI: [0.000, 0.125]). The residuals of the correlations in this analysis are displayed in Table 3. Moreover, the standardized estimates and *R*^2^ of this model are illustrated in Fig. 1. In addition, the standardized and unstandardized estimates are shown in Table 4.

**Table 3:** Residuals of the path analysis correlations with the total Ruminative Responses Scale score as the dependent variable

	1	2	3	4	5	6
1. Rumination total	0.000					
2. Negative interpersonal events	0.077	0.000				
3. Negative achievement events	0.000	0.015	0.000			
4. Aggressive behaviors	0.000	0.008	0.002	0.000		
5. Response inhibition deficits	0.000	0.000	0.000	0.000	0.000	
6. Attentional inhibition deficits	0.001	0.001	0.000	0.000	0.000	0.000

**Table 4:** Path analysis’s unstandardized and standardized estimates with Ruminative Responses Scale’s total score as the dependent variable

Paths	Unstandardized estimates			Standardized estimates		
	*b*	SE	95% CI	*β*	SE	95% CI
Response inhibition deficits to aggressive behaviors	0.126	0.054	[0.021, 0.232]	0.196	0.079	[0.033, 0.344]
Response inhibition deficits to negative interpersonal events	0.103	0.045	[0.010, 0.189]	0.178	0.079	[0.014, 0.325]
Response inhibition deficits to negative achievement events	0.103	0.048	[0.014, 0.199]	0.181	0.082	[0.021, 0.338]
Response inhibition deficits to rumination total	0.172	0.088	[0.011, 0.346]	0.155	0.077	[0.010, 0.304]
Attentional inhibition deficits to aggressive behaviors	− 0.037	0.060	[− 0.151, 0.088]	− 0.044	0.072	[− 0.189, 0.097]
Attentional inhibition deficits to negative interpersonal events	− 0.013	0.065	[− 0.139, 0.116]	− 0.017	0.085	[− 0.182, 0.149]
Attentional inhibition deficits to negative achievement events	0.044	0.066	[− 0.080, 0.180]	0.058	0.088	[− 0.106, 0.240]
Attentional inhibition deficits to rumination total	− 0.064	0.098	[− 0.261, 0.125]	− 0.045	0.067	[− 0.178, 0.086]
Aggressive behaviors to negative interpersonal events	0.154	0.063	[0.035, 0.285]	0.169	0.065	[0.039, 0.296]
Negative interpersonal events to rumination total	0.534	0.144	[0.246, 0.810]	0.281	0.075	[0.126, 0.422]
Negative achievement events to rumination total	0.210	0.157	[− 0.121, 0.490]	0.108	0.083	[− 0.059, 0.263]

Response inhibition deficits were significantly and positively associated with aggressive behaviors, negative interpersonal events, negative achievement events, and rumination. On the other hand, attentional inhibition deficits were not significantly associated with aggressive behaviors, both negative events, and rumination. Aggressive behaviors were positively associated with negative interpersonal events, and negative interpersonal events were positively associated with rumination.

A bias-corrected bootstrap test was conducted to determine the significance of the hypothesized indirect effects. The results indicated significant indirect effects from response inhibition deficits to aggressive behaviors to negative interpersonal events to rumination (*b* = 0.010, *SE* = 0.007, *β* = 0.009, 95% CI: [0.001, 0.029]).

We also conducted a path analysis with depression as an exogenous variable influencing aggressive behaviors, stressors, and rumination. This model had a good fit for the data (*χ*^2^ (2) = 1.751, *p* = 0.417; CFI = 1.000; RMSEA = 0.000, 90% CI: [0.000, 0.130]). The residuals of the correlations in this analysis ranged from − 0.006 to 0.072. Results showed that deficits in response inhibition had a significant positive association with aggressive behaviors (*b* = 0.120, SE = 0.055, *β* = 0.187, *p* = 0.020, 95% CI: [0.023, 0.340]) but not with negative interpersonal events (*b* = 0.059, *SE* = 0.041, *β* = 0.101, *p* = 0.158, 95% CI: [− 0.045, 0.236]) and achievement events (*b* = 0.053, *SE* = 0.049, *β* = 0.093, *p* = 0.275, 95% CI: [− 0.074, 0.261]). In addition, negative interpersonal events were positively associated with rumination (*b* = 0.312, *SE* = 0.129, *β* = 0.165, *p* = 0.017, 95% CI: [0.023, 0.292]), whereas response inhibition deficits, attentional inhibition deficits, and negative achievement events did not show significant associations with rumination (− 0.131 ≤ *b*s ≤ 0.135, − 0.090 ≤ *β*s ≤ 0.122, *p*s ≥ 0.054). Each variable explained 3.9% of the variances in aggressive behaviors, 23.6% of the variances in negative interpersonal events, 25.2% of the variances in negative achievement events, and 31.6% of the variances in rumination. Bootstrap tests indicated the 95% CI of the indirect effect from response inhibition deficits to aggressive behaviors to negative interpersonal events to rumination included zero (*b* = 0.005, SE = 0.004, *β* = 0.005, 95% CI: [0.000, 0.017]).

Next, we analyzed the brooding subscale score instead of the total RRS score as the dependent variable. The model provided an excellent fit to the data (*χ*^2^ (2) = 1.434, *p* = 0.489; CFI = 1.000; RMSEA = 0.000, 90% CI: [0.000, 0.123]). The residuals of the correlations in this analysis ranged from 0.000 to 0.075. Negative interpersonal events showed significant positive associations with brooding (*b* = 0.184, *SE* = 0.045, *β* = 0.318, *p* < 0.001, 95% CI: [0.166, 0.455]), whereas other variables including response inhibition deficits did not show significant associations with brooding (− 0.022 ≤ *b*s ≤ 0.051, − 0.050 ≤ *β*s ≤ 0.108, *p*s ≥ 0.143). Each variable explained 16.6% of the variances in brooding. Bootstrap tests indicated significant positive indirect effect from response inhibition deficits to aggressive behaviors to negative interpersonal events to brooding (*b* = 0.004, SE = 0.002, *β* = 0.011, 95% CI: [0.002, 0.031]).

We repeated the identical analysis using the reflection subscale score instead of the brooding subscale score. The model provided an excellent fit to the data (*χ*^2^ (2) = 1.357, *p* = 0.508; CFI = 1.000; RMSEA = 0.000, 90% CI: [0.000, 0.121]). The residuals of the correlations in this analysis ranged from 0.000 to 0.075. Results showed that neither variable was significantly related to reflection (− 0.022 ≤ *b*s ≤ 0.075, − 0.060 ≤ *β*s ≤ 0.158, *p*s ≥ 0.069). Each variable explained 5.4% of the variances in reflection. Bootstrap tests showed the 95% CI of the indirect effect from response inhibition deficits to aggressive behaviors to negative interpersonal events to reflection included zero (*b* = 0.001, SE = 0.001, *β* = 0.005, 95% CI: [0.000, 0.022]).

## Discussion

This study indicated that response inhibition deficits had an indirect positive association with rumination via increases in aggressive behaviors and negative interpersonal events. The significant positive relationship between response inhibition deficits and aggressive behaviors is consistent with previous findings (Qiao et al., 2016; Raaijmakers et al., 2008), and suggest that individuals with response inhibition impairments have a reduced ability to control aggressive behaviors. Results also suggested that negative interpersonal events increased as a result of aggressive behaviors leading to rumination, which was not the case with negative achievement events, although caution is needed in making this interpretation because differences between simple correlation coefficients of negative interpersonal events with rumination and negative achievement events with rumination were small. Nevertheless, the finding that negative interpersonal events are primarily related to rumination is consistent with the psychobiological theory of depression proposed by Slavich et al. (2010). They suggested that because humans, who are social animals, have a fundamental drive to maintain positive social status, social values, and social regard, social rejections that threaten these needs might elicit negative self-referential cognitions concerning social worth and esteem (i.e., rumination). This could be the reason why only negative interpersonal events, mostly consisting of rejection events, were positively related to rumination.

Associations between each of the variables described above remained significant even after controlling for the influence of depression level, although caution is needed because the indirect effect from response inhibition deficits to rumination via increases in aggressive behaviors and negative interpersonal events was not significant. These results indicated that depression might not be a confounding factor in the relationship among these variables. On the other hand, the significant direct associations between response inhibition deficits and the two types of negative events disappeared after controlling for depression. These findings suggest that response inhibition deficits might be positively related to rumination through interpersonal processes.

Inconsistent with the study by Altamirano et al. (2010), the current study indicated that attentional inhibition deficits were unrelated to trait rumination. A nonsignificant association between attentional inhibition deficits and trait rumination was also reported in Nishimura et al. (2020). Therefore, it is implausible that high ruminators could better resist interference from distracting stimuli. On the other hand, the incongruent-trial error rates in the MST were correlated with increased depressive symptoms, which is consistent with the finding by Altamirano et al. (2010), although it should be noted that the significant correlation disappeared when the data of participants with multivariate outliers were excluded (see “Statistical analysis”). These results might reflect symptoms of impaired concentration in dysphoric and depressed individuals (American Psychiatric Association, 2013; Beck et al., 1996).

The findings of this study suggest that response inhibition and attentional inhibition could be discriminated from each other based on their relationship with trait rumination because only response inhibition was significantly associated with rumination, and the magnitude of the correlation with rumination differed significantly between the two types of inhibition. A previous study using confirmatory factor analysis has also indicated that response inhibition and attentional inhibition could be discriminated (Tiego et al., 2018). Recent studies have emphasized that the latent variable named common executive function––composed of performance in tasks assessing inhibition, updating, and shifting––predicts trait rumination (du Pont et al., 2019; Gustavson et al., 2020; Snyder et al., 2019). However, we propose that subdivisions of inhibition might play a crucial role in the background to rumination.

It is possible that the significant relationship between response inhibition and trait rumination was caused by the shared variance in working memory capacity, which reflects the ability to actively maintain a goal in working memory. Tiego et al. (2018) used confirmatory factor analysis and indicated that working memory capacity is a distinct construct from response inhibition and attentional inhibition, and the two inhibition subcomponents are dependent on working memory capacity. We did not assess working memory capacity, and therefore, could not examine whether this ability might be a confounding factor in the association between response inhibition and rumination, although this is unlikely because a previous study has indicated that working memory capacity assessed by the Operation Span Task had a significant *positive* association with brooding (Onraedt & Koster, 2014). We suggest that future studies compare associations of rumination with response inhibition, attentional inhibition, and working memory capacity, after controlling for the influences of other variables.

In line with the total RRS score analysis, the indirect effect from response inhibition deficits to the brooding subscale scores via increases in aggressive behaviors and negative interpersonal events was significant. On the other hand, the indirect effect from response inhibition deficits to reflection was not significant and was weaker than brooding. The magnitudes of these indirect effects were due to different relationships between negative interpersonal events and each rumination subcomponent. The finding that brooding was more strongly related to interpersonal stressors than reflection is consistent with Hasegawa et al. (2021). These results also supported Slavich et al. (2010), who suggested that social rejection leads to negative self-referential thoughts concerning social worth and self-esteem, which largely overlap with brooding. The reduced negative valence of reflection might explain the weaker association between reflection and negative interpersonal events than the association between brooding and negative interpersonal events.

Results of the current study indicated that neither response inhibition nor attentional inhibition was significantly correlated with trait worry, which contrasted with the significant association between response inhibition and rumination. However, differences between rumination and worry in the magnitude of correlation coefficients with response inhibition were small and statistically nonsignificant, which supported the findings of a meta-analysis reporting that executive functions were not differentially related to rumination or worry (Zetsche et al., 2018). The nonsignificant difference between rumination and worry in the magnitude of the correlation with response inhibition might have resulted from low statistical power. In addition, Watson et al. (2017) indicated that the PSWQ is not a specific measure of anxiety-related constructs and that it is better considered a general measure of neuroticism. Therefore, we suggest that the current study be replicated in the future using a more specific measure of worry.

There are some limitations in this study in addition to the issue discussed above. First, the sample consisted only of university students, making it unclear whether the study’s findings are generalizable to other age groups or clinical samples. In addition, the post hoc power of the correlation analyses indicated that larger sample size was required for detecting the hypothesized association (see Footnote 5). Future studies are needed to replicate these findings in different and larger samples. Second, the cross-sectional design of the study precludes identifying causal relationships, which require a longitudinal design. Third, this study used single tasks to measure response inhibition and attentional inhibition rather than latent variables constructed from multiple task performance. Executive function tasks have a task-impurity problem and low test–retest reliability, and therefore, a latent variable approach is ideal for examining relationships between executive functions and other variables of interest, including psychopathological symptoms (Snyder et al., 2015). Future studies should adopt a latent variable approach for examining associations between each subcomponent of inhibition and trait rumination and worry. Finally, this study did not use a scale dedicated to assessing dependent stressors that are generated by individuals with maladaptive characteristics (Hammen, 1991; Liu & Alloy, 2010), because scales for separately assessing independent and dependent stressors are unavailable in Japan. It is suggested that future studies develop a measure for separately evaluating independent and dependent stressors and examine whether response inhibition increases rumination via an increase in dependent interpersonal stressors, regardless of dependent achievement stressors and independent stressors.

## Data Availability

This study’s dataset can be found at the Open Science Framework [https://osf.io/8hfzt/].

## Footnotes

1 Nearly the same results as those described below were obtained using a latent variable constructed from the commission error rates in four GNG blocks.

2 The CCPT is conducted to examine the appropriate inter-stimulus interval of the GNG for measuring response inhibition (see Hasegawa et al., 2020). Although the CCPT is another task assessing response inhibition, the performance in the CCPT seems to reflect other factors such as inattention because the CCPT task takes longer to complete. Therefore, GNG performance might be a better index for assessing response inhibition than CCPT. In addition, it is reasonable to compare associations of response inhibition and attentional inhibition with other variables under identical conditions at the single task level. Therefore, we did not use the CCPT in the analysis.

3 We used the BAQ to explore the association between two subcomponents of inhibition and each subcomponent of aggression. The BAQ seems to be an inappropriate measure of aggressive behaviors because this scale includes specific physical aggression items that university students rarely conduct, such as violent behaviors, and does not include items on offensive language use. Therefore, we did not use the BAQ in this study.

4 We have reported all data exclusions and all the measures used in this study.

5 We computed the post hoc power of correlations obtained with R. This study specifically examined the indirect effects of response inhibition to aggressive behaviors to negative interpersonal events to rumination assessed by the total RRS score. The correlation between response inhibition and aggressive behaviors was the smallest (*r *= 0.185) among simple correlations between these variables. We had 77.6% power set at *α *= 0.05, two-tailed, to detect this correlation coefficient.
