# Correction to: Response inhibition deficits are positively associated with trait rumination, but attentional inhibition deficits are not: aggressive behaviors and interpersonal stressors as mediators

**DOI:** 10.1007/s00426-021-01582-7

**Published:** 2021-11-01

**Authors:** Akira Hasegawa, Noboru Matsumoto, Yuko Yamashita, Keisuke Tanaka, Jun Kawaguchi, Tetsuya Yamamoto

**Affiliations:** 1 Faculty of Human Relations, Tokai Gakuin University, 5-68 Naka-kirino, Kakamigahara City, Gifu 504-8511 Japan; 2 Division of Psychology, Faculty of Arts, Shinshu University, Asahi 3-1-1, Matsumoto, Nagano 390-8621 Japan; 3 Mutsumi Hospital, 3-11-23, Minamiyaso-cho, Tokushima, 770-0005 Japan; 4 Graduate School of Education, Joetsu University of Education, 1-Yamayashiki-machi, Joetsu-shi, Niigata, 943-8512 Japan; 5 Department of Psychology, Otemon Gakuin University, 2-1-15, Nishiai, Ibaraki City, Osaka 567-8502 Japan; 6 Graduate School of Informatics, Nagoya University, Furo-cho, Chikusa-ku, Nagoya, Aichi 464-8601 Japan; 7 Graduate School of Technology, Industrial and Social Sciences, Tokushima University, 1-1, Minamijosanjima-cho, Tokushima, 770-8502 Japan

## Correction to: Psychological Research 10.1007/s00426-021-01537-y

In the original publication of the article, the information listed in Table 2 was not formatted correctly (1) all variables (e.g., “Depression”) are written in one or two lines, and (2) 95% confidence intervals are not broken (i.e., written in one line; e.g., [0.663, 0.790]). Hence this has been corrected and published in the erratum article as given below:

**Table 2:** Correlations between variables

	1	2	3	4	5	6	7	8	9
1. Depression	−								
2. Brooding	0.439^***^	−							
	[0.330, 0.547]								
3. Reflection	0.221^**^	0.477^***^	−						
	[0.093, 0.349]	[0.373, 0.581]							
4. Rumination total	0.526^***^	0.874^***^	0.726^***^	−					
	[0.429, 0.624]	[0.842, 0.906]	[0.663, 0.790]						
5. Worry	0.459^***^	0.523^***^	0.288^***^	0.540^***^	−				
	[0.352, 0.565]	[0.425, 0.622]	[0.164, 0.411]	[0.443, 0.636]					
6. Negative interpersonal events	0.441^***^	0.387^***^	0.198^**^	0.374^***^	0.271^***^	−			
	[0.333, 0.550]	[0.272, 0.502]	[0.069, 0.328]	[0.257, 0.490]	[0.147, 0.395]				
7. Negative achievement events	0.492^***^	0.274^***^	0.144^*^	0.290^***^	0.281^***^	0.564^***^	−		
	[0.390, 0.595]	[0.148, 0.400]	[0.012, 0.276]	[0.166, 0.414]	[0.157, 0.406]	[0.471, 0.656]			
8. Aggressive behaviors	0.083	0.159^*^	0.130	0.165^*^	− 0.077	0.211^**^	0.041	−	
	[− 0.052, 0.218]	[0.027, 0.291]	[− 0.002, 0.262]	[0.034, 0.297]	[− 0.211, 0.056]	[0.081, 0.341]	[− 0.098, 0.179]		
9. Response inhibition deficits	0.220^**^	0.178^**^	0.145^*^	0.225^**^	0.112	0.204^**^	0.195^**^	0.185^**^	−
	[0.092, 0.348]	[0.048, 0.308]	[0.013, 0.276]	[0.097, 0.352]	[− 0.021, 0.245]	[0.076, 0.333]	[0.066, 0.325]	[0.054, 0.316]	
10. Attentional inhibition deficits	0.174^**^	− 0.004	− 0.020	0.021	0.063	0.028	0.098	− 0.006	0.266^***^
	[0.043, 0.305]	[− 0.141, 0.132]	[− 0.159, 0.118]	[− 0.118, 0.160]	[− 0.071, 0.198]	[− 0.108, 0.164]	[− 0.038, 0.234]	[− 0.154, 0.143]	[0.138, 0.393]

Numbers in parentheses indicate 95% confidence intervals. * *p* < 0.05, ** *p* < 0.01, *** *p* < 0.001

